# A lexicon based method to search for extreme opinions

**DOI:** 10.1371/journal.pone.0197816

**Published:** 2018-05-25

**Authors:** Sattam Almatarneh, Pablo Gamallo

**Affiliations:** Centro Singular de Investigación en Tecnoloxías da Información (CITIUS), University of Santiago de Compostela, Santiago de Compustela, A Coruña, Spain; Nanyang Technological University, SINGAPORE

## Abstract

Studies in sentiment analysis and opinion mining have been focused on many aspects related to opinions, namely polarity classification by making use of positive, negative or neutral values. However, most studies have overlooked the identification of extreme opinions (most negative and most positive opinions) in spite of their vast significance in many applications. We use an unsupervised approach to search for extreme opinions, which is based on the automatic construction of a new lexicon containing the most negative and most positive words.

## Introduction

After the massive explosion in the use of the Internet and social media in various aspects of life, social media has come to play a significant role in guiding people’s tendencies in social, political, religious and economic domains, through the opinions expressed by individuals. In the last decade, a huge number of studies have been carried in the field of opinion mining and sentiment analysis.

The fundamental task in Opinion Mining is polarity classification [1–3], which occurs when a piece of text stating an opinion is classified into a predefined set of polarity categories (e.g., positive, neutral, negative). Reviews such as “thumbs up” versus “thumbs down”, or “like” versus “dislike” are examples of two-class polarity classification. An unusual way of performing sentiment analysis is to detect and classify extreme opinions, which represent the most negative and most positive opinions about a topic, an object or an individual. An extreme opinion is the worst or the best view, judgment, or appraisal formed in ones mind about a particular matter.

**Fig 1:**
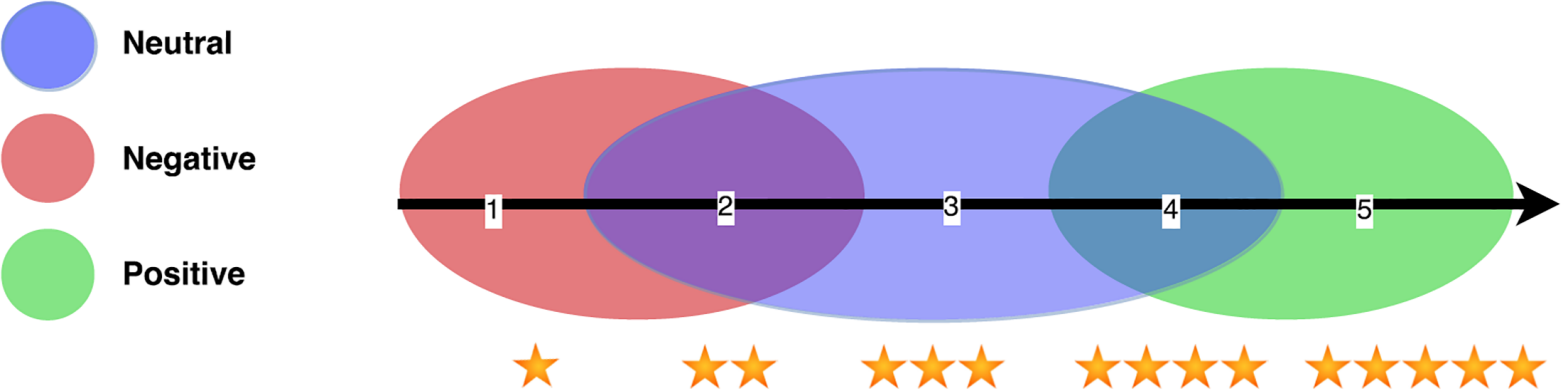
Red Hypothetical continuous distribution of negative, neutral and positive views on a scale from 1 to 5, according to the borderline between stars.

One of the main motivations for detecting extreme opinions is the fact that they actually stand for *pure* positive and negative opinions. As rating systems have no clear borderlines on a continuum scale, weakly polarized opinions (e.g. those rated as 4 and 2 in a 1 to 5 rating system) may be in fact closer to neutral statements. According to Pang and Lee [4], “it is quite difficult to properly calibrate different authors’ scales, since the same number of *stars* even within what is ostensibly the same rating system can mean different things for different authors”. Given that rating systems are defined on a subjective scale, only extreme opinions can be seen as natural, transparent, and non ambiguous positive / negative statements. Fig 1 shows the spread of negative, neutral and positive opinions on a scale from 1 to 5. Red, blue, and green colors stand for negative, neutral and positive opinions, respectively. Color overlap covers the space around 2 and 4, where neutral views may appear together with light negative and positive opinions. Pure red and green appear only around 1 and 5 stars, the extreme opinions.

Extreme opinions only constitute a small portion of the opinions on Social Media. According to [4], only about 5% of all opinions are on the most extreme points of a scale, which makes the search for these opinions a challenge. We are then confronted with a challenging task. The literature on Opinion Mining and Sentiment Analysis has mostly ignored extreme opinions in spite of their importance if the objective is to identify the most relevant weaknesses and strengths of each product or organization from the viewpoint of customers. The most negative viewpoints help identify what the most annoying aspects of products for customers are and what the defective goods are. On the other hand, strongly positive views allow for the identification and selection of outstanding products, services and sellers.

Also, extreme views may be indicative of fraud practiced by some organizations, namely when they write very positive online reviews about themselves to raise their rating. Similarly, these extreme reviews are also used to discredit a product or service, since some competitors may write very negative reviews to reduce the sales of their competitors as a kind of unfair competition, as mentioned in [5].

It is not surprising that extreme views have a strong impact on product sales, since they influence customer decisions before buying. Previous studies analyzed this relationship, such as the experiments reported in [6], which found that as the high proportion of negative online consumer reviews increased, the consumer’s negative attitudes also increased. Similar effects have been observed in consumer reviews: one-star reviews significantly hurt book sales on Amazon.com [7]. The impact of 1-star reviews, which represent the most negative views, is greater than the impact of 5-star reviews in this particular market sector.

Last but not least, another motivation for the identification of extreme opinions is the current use of bot technology by cyborgs on social networks. These bots are designed to sell products or attract clicks, amplifying false or biased stories in order to influence public opinion.

We consider there is a need for systematic studies attempting to understand how to mine the vast amount of unstructured text data in order to extract extreme comments and opinions. Most previous studies have considered that, in whatever rating system, it is possible to identify three categories: negative, neutral, and positive views. For instance, on a 5-rating scale, negative opinions are those that belong to the reviews of one and two stars, the positive views are those assigned four and five-star reviews, while three-star is neutral. By contrast, our study relies on two binary classification tasks focused on identifying extreme opinions. First, we build a classifier identifying the most negative views against other opinions, including not very negative, neutral, and positive. Secondly, we also define a classifier, selecting the most positive views from the rest of opinions, namely those that are not very positive, neutral, and negative. The key aspect of our strategy is based on the construction of the polarity lexicon underlying classification.

More precisely, the main contribution of this article is to investigate the effectiveness of the automatic construction of a sentiment lexicon using unsupervised machine learning classification to search for extreme opinions. This is the first step towards improving mining tools in various domains (e.g., e-commerce, industry, politics, etc.). Our experiments will be carried out using reviews on commercial products and movies. There are, at least, two types of strategies for sentiment analysis: Machine-Learning-Based and lexical-based. Machine learning strategies usually rely on supervised classification which tends to detect the sentiment in binary terms (i.e., positive or negative). This approach needs labeled text data to train classifiers. The main drawback is the scarce availability of labeled data in many domains and hence the low applicability of the method to new data and new domains [8]. This is the case with our study since we only have a limited number of available scaled dataset that might be classified into two classes: the most negative *vs.* others or the most positive *vs.* others. In our previous study [9], we made an exhaustive study of the effectiveness of linguistic features in supervised machine learning classification to search for the most negative opinions. The experiments we reported on that work showed low performance for all configuration systems. This means that the task of searching for extreme opinions is very challenging even for supervised strategies.

Unsupervised machine learning does not require a sufficient amount of human-annotated training data to obtain acceptable results. This has motivated us to look for methods that do not need training data or need only a relatively small amount of it. The most popular unsupervised strategies used in sentiment analysis are lexical-based methods. They make use of a predefined list of words, where each word is associated with a specific sentiment. Lexicon-based strategies are very efficient and simple methods. They make use of a sentiment lexicon to assign a polarity value to each text document by following a basic algrithm. A sentiment lexicon is a list of lexical features (e.g., words, phrase, etc.) which are labeled according to their semantic orientation (i.e. polarity) as either positive or negative [10].

There are three main ways of building sentiment lexicons: hand-craft elaboration, [11, 12], automatic expansion from an initial list of seed words [13–15], and corpus-based approaches [16]. Corpus-based approaches also make use of a list of seed sentiment words to find other sentiment words and their polarity from the given corpus.

In this article, our main objective is to describe a corpus-based method to build an opinion lexicon by distinguishing the most negative and most positive terms from the other opinion words. In addition, the terms of the lexicon are weighted and ranked from the most negative values to the least negative ones, and from the most positive to the least positive values.

As a result, a new sentiment lexicon has been developed with the aid of the corpus collected by [17, 18]. The new lexical resource is used by sentiment analysis classifiers to find extreme opinions. This specific task will allow us to evaluate the quality of the new lexical resource by comparing it to other sentiment dictionaries.

The rest of the paper is organized as follows. In the following section (Section two) we describe the related work. Then, Section three describes the method used to create our proposed lexicon and how to use it in the classification task. Experiments are introduced in Section four, where we also describe the evaluation and discuss the results. We draw the conclusions and discuss future work in the last section.

## Related work

One of the pioneer studies describing a corpus-based method to determine the orientation or polarity of adjectives was reported in [16]. The method was unsupervised and relied on a basic linguistic assumption: adjectives co-occurring with conjunctions in a corpus are provided with the same polarity, namely positive or negative. Since then, many other unsupervised strategies were inspired by the corpus-based method reported in that article. A simple unsupervised learning algorithm was presented by [13], who classified reviews into two categories “recommend or not recommend” depending on the average number of positive and negative phrases which appeared in the review.

Their algorithm consists of the following steps: first, it searches for phrases in the review by using a Part-Of-Speech (POS) tagger and then determines the polarity of the extracted phrases by computing Pointwise Mutual Information and Information Retrieval (PMI-IR). Then, the algorithm identifies those associative words returned by the search engine using the NEAR operator. Finally, the polarity of each phrase is determined by computing all the polarities returned by the search engine. In contrast, [19] implemented a completely supervised machine learning method to classify a whole review as positive or negative.

The work by [15] was focused on the use of the synonymy relation between adjectives in WordNet [20] to generate a graph. The strategy measures the shortest path between the adjective and two basic sentiment seeds, “good” and “bad”, to determine the polarity of a word. This is a semi-supervised learning method which starts with a lexical resource, WordNet, and a small list of seeds in order to expand the lexical resource in an iterative process.

Other research conducted by [21] suggests a method for extracting polarity for phrases. They build lexical networks connecting similar words with two types of links: words linked with the same polarity and those with different polarity. The proposed method can classify adjective-noun phrases consisting of unseen words.

[22] proposed a holistic lexicon-based approach which improved the lexicon-based method proposed by [14]. Their approach solved the context-dependent problem of opinion words by utilizing information from other sentences rather than looking at only one sentence. This strategy takes some linguistic properties of natural language expressions into account in order to infer the polarity of opinion words. It requires no prior domain knowledge or user inputs. The authors also propose a solution for the problem of having multiple conflicting opinion words in a sentence, by considering the distance between each opinion word and the product feature. [23] proposed an approach to find the polarity of reviews by converting text into numeric matrices using *countvectorizer* and TF-IDF, and then using it as input in machine learning algorithms for classification.

A lot of different research has been conducted in this area recently with different directions. [24] proposed a novel paradigm to concept-level sentiment analysis that merges linguistics, common-sense computing, and machine learning for improving the accuracy of polarity detection. [25] also introduced a brain-inspired sentiment analysis framework for real-time concept-level research to help machines emulate human inference of sentiment from natural language.

More precisely, the introduced approach combines the use of linguistic patterns based on the syntactic structure of the sentences. The algorithm defines the polarity of each word and flows or extends it through the dependency arcs to determine the final polarity label of the sentence. [26] proposes models relying on domain-dependent opinions and use latent variables instead of words or phrases to classify sentiments. In [27], opinions are inferred by using an algorithm based on spectral optimization of a modularity matrix. [28] suggests methods for detecting noun words that are perceived as being objective (without polarity) even if they also imply opinions. Another promising piece of work introduced by [29] aims to discover contradicting opinions in blogs, which can be useful in tracking opinion evolution over time. [30] proposed a lexicon-enhanced method for improving the sentiment analysis of user generated reviews based on a rule-based classification scheme. [31] built a lexicon containing a combination of sentiment polarity (positive, negative) with one of eight possible emotion classes (anger, anticipation, disgust, fear, joy, sadness, surprise, trust) for each word. [32] proposed a cross-language opinion lexicon extraction framework using the mutual-reinforcement label propagation algorithm. [33] proposed a semi-supervised framework for generating a domain-specific sentiment lexicon to reduce human effort for constructing a high quality domain-specific sentiment lexicon. Also in recent years many studies in sentiment analysis started working on the deep learning paradigm, such as [34, 35]. In this sense, [36] presented a new method to identify sentiment polarity in video clips of people speaking. They used deep Convolutional Neural Networks to extract features from text and feed them into multi-kernel to classify the multimodal heterogeneous fused feature vectors. Although several opinion lexicons containing the polarity and the strength of words have been built [11, 12, 37–40], they are not focused on the most negative and most positive words. We propose a new method to build opinion lexicons from multiple domains for the most negative and most positive words, which is quite a different resource with regard to existing lexicons. As far as we know, no previous work has been focused on detecting extreme opinions. Our proposal, therefore, may be considered to be a first step in that direction.

## The method

Our strategy consists of two tasks: first, we create a corpus-based polarity lexicon with two values for each case: very negative and not very negative, on the one hand, and very positive and not very positive, on the other. Secondly, sentiment classification is carried out on the basis of this lexical resource. The data collected from websites are publicly available data, and no personally identifiable information of the users was gathered, and we complied with all the terms and conditions of service of the websites that we used in this study. All datasets are described in the *Test Dataset* Section.

### Automatic construction of polarity lexicons

We describe how to build two lexicons: one that ranks words on the negative scale, from the most negative values to the least negative ones, and another lexicon in the positive domain, which arrange values from the most positive to the least positive. The lexicons can be generated using any corpus of reviews labeled with a star rating: one star (most negative) to N stars (most positive). The category set is the number of stars that can be assigned to the reviews. For instance, we are provided with 10 categories only if each review can be rated from 1 to 10.

The first step to create our proposed lexicons is to measure the relative frequency (RF) for every word *w* in each category *c* according to Eq 1:

$$RF_{c}\left(w\right)=\frac{freq(w,c)}{Total_{c}}$$ (1)

where *c* is any category of the star rating, from 1 to *N*; *freq*(*w*, *c*) is the number of tokens of the target word in *c*; and *Total*_*c*_ is the total number of word tokens in *c*. As in our experiments the corpus was PoS tagged, words are actually represented as (word, tag) pairs. Besides, we only work with adjectives and adverbs as they are the most relevant part of speech in sentiment analysis for any language, according to [41, 42].

The second step is to calculate the average of RF values for two ranges of categories: most negative (MN) *vs* not most negative (NMN), and most positive (MP) *vs* not most positive (NMP). For this purpose, it is necessary to define a borderline value *B* for extreme opinions, which might vary according to the specific star rating of the reviews. For instance, if the rating goes from 1 to 10, and the borderline value B = 2, the MN reviews are considered those rated from 1 to 2, while MP are those rated from 8 to 10. This is similar if the rating goes from 1 to 5 and the borderline is set at 1. In this case, the MN reviews are considered those rated 1, while MP are those rated 5. Given a borderline value, *B*, the average of the MN scores, *AvMN*, for a word is computed as follows:

$$AvMN\left(w\right)=\frac{\sum_{c=1}^{B}RF_{c}\left(w\right)}{B}$$ (2)

On the other hand, given *R* = *N* − *B*, where *N* is the total number of categories, the average of NMN values, *AvNMN*, for each word is computed in Eq 3:

$$AvNMN\left(w\right)=\frac{\sum_{c=B+1}^{N}RF_{c}\left(w\right)}{R}$$ (3)

As for the average of MP scores, *AvMP*, for a word, it is computed in Eq 4:

$$AvMP\left(w\right)=\frac{\sum_{c=(N+1)-B}^{N}RF_{c}\left(w\right)}{B}$$ (4)

And the average of NMP values, *AvNMP*, for each word is computed in Eq 5:

$$AvNMP\left(w\right)=\frac{\sum_{c=1}^{N-B}RF_{c}\left(w\right)}{R}$$ (5)

In the following step, the objective is to assign polarity weights to words and classify them by using four polarity classes: MN, NMN, MP, and NMP. Extreme words (MN and MP) are separated from not extreme words by just comparing the difference between the average values obtained by the equations defined above: 2, 3, 4, 5. With this simple idea, we build two lexicons: one lexicon on the negative scale from MN to NMN, and another lexicon on the positive scale from MP to NMP. So, given a word *w*, we compute the differences *D*_*neg*_ and *D*_*pos*_ in Eqs 6 and 7, and assign the resulting values to *w*:

$$D_{neg}\left(w\right)=AvNMN\left(w\right)-AvMN\left(w\right)$$ (6)

$$D_{pos}\left(w\right)=AvNMP\left(w\right)-AvMP\left(w\right)$$ (7)

*D*_*neg*_ gives a weight to *w* within the negative scale, while *D*_*pos*_ assigns weights in the positive ranking. These two weights are used to classify words in the four aforementioned categories and thereby building two new polarity lexicons, which we call *VERY-NEG* and *VERY-POS*. Classification is carried out with the following basic algorithm:

If the value of *D*_*neg*_(*w*) is negative, *w* is in the MN class. If *D*_*neg*_(*w*) is positive, *w* is in NMN.

If the value of *D*_*pos*_(*w*) is positive, *w* is in the MP class. If *D*_*pos*_(*w*) is negative, *w* is in NMP.

VERY-NEG is a lexicon made up of words classified as MN or NMN, while VERY-POS is another lexicon consisting of words classified as MP or NMP. In both lexicons, words are ranked by means of the weight returned by *D*_*neg*_ or *D*_*pos*_.

### Sentiment classification

**Fig 2:**
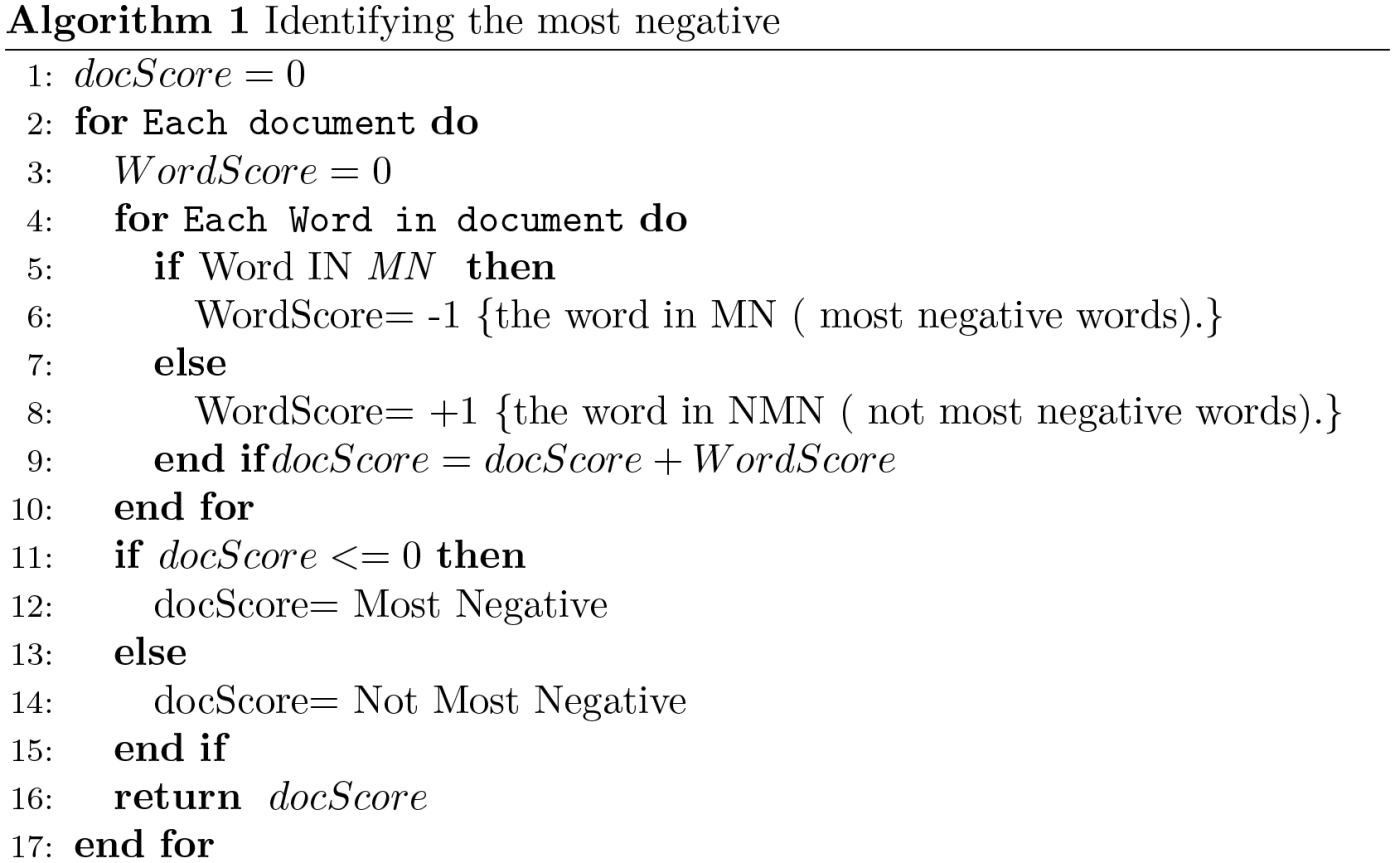
Algorithm to assign the most negative classification to an input document.

**Fig 3:**
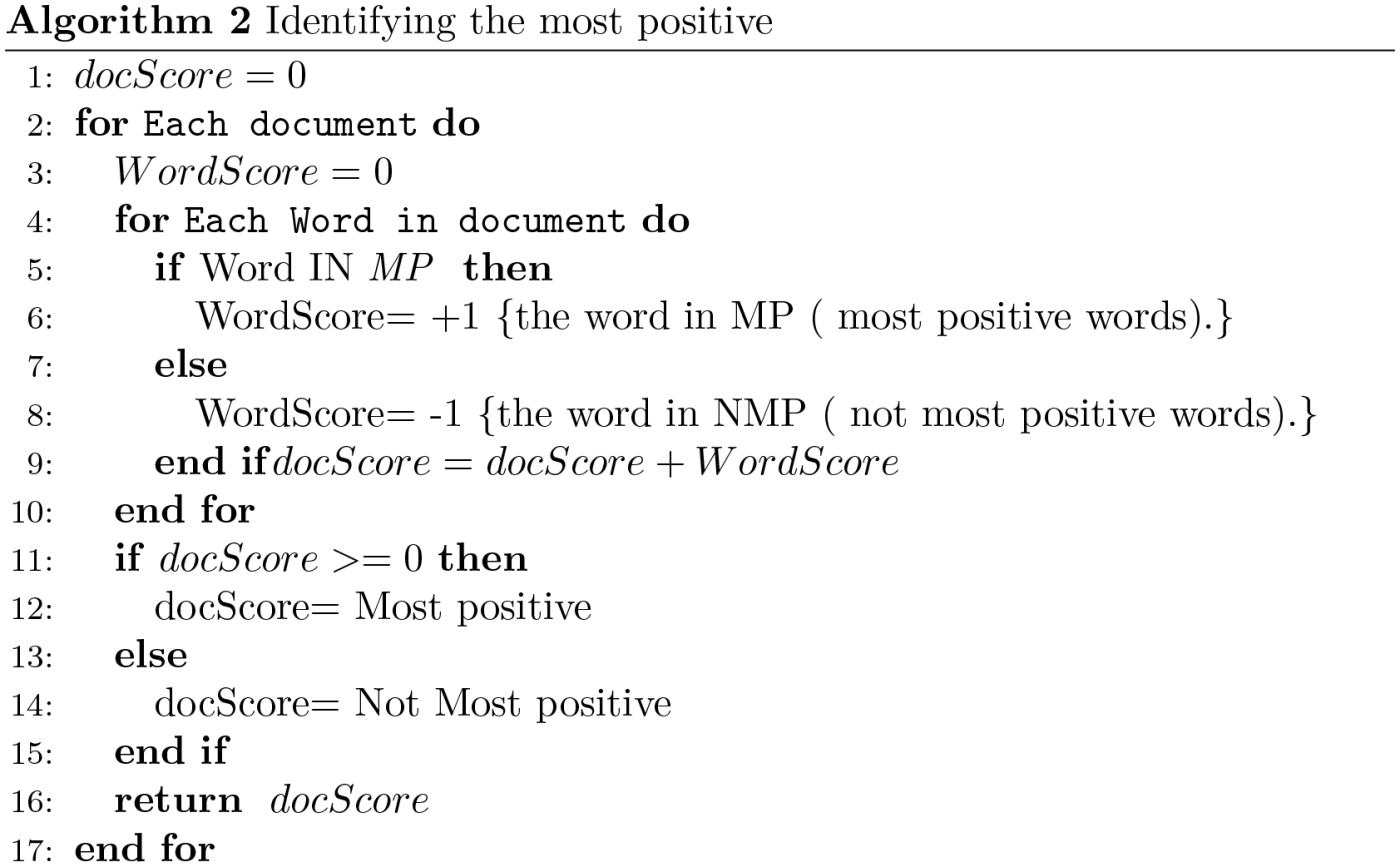
Algorithm to assign the most positive classification to an input document.

Sentiment analysis typically works at three levels of granularity, namely, document level, sentence level, and aspect level. We are involved with document-level classification and two polarity classes: extreme *vs.* non-extreme opinions. Sentiment classification is carried out as follows. First, a part-of-speech tagger is applied to extract adjectives and adverbs from reviews. Then, the algorithm plotted in Figs 2 and 3 is applied. This is a basic word-matching scheme to carried out unsupervised sentiment classification. In particular, the sentiment polarity of a word is obtained from the sentiment lexicon built in the previous step. In the case of classification between MN and NMN, the algorithm in Fig 2 assigns -1 to MN words and +1 to NMN. On the other hand, in the case of classification between the MP and NMP, the algorithm assigns +1 to MP words and -1 to NMP as in Fig 3.

The overall sentiment score of a document is simply computed as the sum of the sentiment scores of the words in the document.

## Experiments

In order to cover several domains, the experiments were carried out using different datasets, including books, DVD, electronics, housewares, and movie reviews. In our experiments, we automatically built two polarity lexicons using the strategy defined above in the Subsection *Automatic construction of polarity lexicons*. Our lexicons were evaluated and compared with other existing handcraft lexicons in the task of classifying extreme reviews. For the purpose of evaluation, we used five different datasets. Before defining the evaluation protocol and showing the results, we describe the resources, both lexicons and corpus-based datasets, used in the experiments.

### Lexicons

As mentioned earlier, there are many popular and available sentiment lexicons. However, for the purpose of comparison, we need lexicons with properties according to the following two criteria:

- First, every entry in the dictionary is required to be assigned a PoS tag.
- Second, every entry must be associated with a score according to its polarity strength.

Four lexicons will be compared: the two lexicons we built using our strategy, called VERY-NEG, VERY-POS, a manual resource reported in [11], called SO-CAL, and SentiWords [43].

#### VERY-NEG and VERY-POS

Our proposed lexicons were built from the text corpora introduced in [17, 18]. It is freely available at: https://web.stanford.edu/~cgpotts/data/wordnetscales/wn-asr-multicorpus.csv.zip. The corpora consist of online reviews collected from IMDB, Goodreads, OpenTable and Amazon/Tripadvisor. Each of the reviews in this collection has an associated star rating: one star (most negative) to ten stars (most positive) in IMDB, and one star (most negative) to five stars (most positive) in all the other corpora.

Reviews were tagged using the Stanford Log-Linear Part-Of-Speech Tagger. Then, tags were broken down into WordNet PoS Tags: *a* (adjective), *n* (noun), *v* (verb), *r* (adverb). Words whose tags were not part of those categories were filtered out. The list of selected words was then stemmed.

Table 1 shows the quantitative information of the adjective “bad”, where *Freq* is the total number of tokens of a (word,tag) pair in each category and corpus, while *Total* is the total number of word tokens in each category and corpus (Total values are constant for all words but repeated for each one in order to make processing easier). Then, we compute *AvMN*, *AvNMN*, *AvMP* and *AvNMP* for each word and obtain the weights (*D*_*neg*_(*w*) and *D*_*pos*_(*w*) values) to build the corresponding lexicons for each corpus. Finally, we compute the average of all weights for the same *w* in order to obtain two cross-domain final lexicons (VERY-NEG and VERY-POS). VERY-NEG contains a list of the most negative words (MN) and a list of words that are not classified as most negative (NMN). In the same way, VERY-POS contains two lists: the most positive words (MP) and the other words (NMP). Both lexicons are freely available at: https://github.com/almatarneh/LEXICONS.

**Table 1:** A sample of the collection format for the word (“bad”, *a*) in each category.

Word	Tag	Category	Freq	Total	Corpus
bad	a	1	122232	25395214	IMDB
bad	a	2	40491	11755132	IMDB
bad	a	3	37787	13995838	IMDB
bad	a	4	33070	14963866	IMDB
bad	a	5	39205	20390515	IMDB
bad	a	6	43101	27420036	IMDB
bad	a	7	46696	40192077	IMDB
bad	a	8	42228	48723444	IMDB
bad	a	9	29588	40277743	IMDB
bad	a	10	51778	73948447	IMDB
bad	a	1	2100	3419923	Goodreads
bad	a	2	1956	3912625	Goodreads
bad	a	3	2780	6011388	Goodreads
bad	a	4	2298	10187257	Goodreads
bad	a	5	2119	16202230	Goodreads
bad	a	1	1127	699695	OpenTable
bad	a	2	2595	2507147	OpenTable
bad	a	3	2859	4207700	OpenTable
bad	a	4	2544	7789649	OpenTable
bad	a	5	1905	8266564	OpenTable
bad	a	1	1241	3419923	Amazon/Tripadvisor
bad	a	2	791	3912625	Amazon/Tripadvisor
bad	a	3	870	6011388	Amazon/Tripadvisor
bad	a	4	1301	10187257	Amazon/Tripadvisor
bad	a	5	2025	16202230	Amazon/Tripadvisor

Through preliminary experiments, we found that the best results were obtained by filtering out words with very low weight (*D* <= 0.00000001), which are values close to zero. This means that we filtered out neutral words, i.e. words without polarity.

In order to ensure that all cases are tested, we created lexicons at two different borderline (B) values: B = 1 and B = 2. The former is used to determine extreme values on scales from 1 to 5. More precisely, when B = 1 we mean that 1 (most negative) and 5 (most positive) are the extreme scores. The latter parametrization (B = 2) is used to define extreme values in scales from 1 to 10: in this case, 1 and 2 are extreme values for most negatives, while 9 and 10 represent the class of most positive opinions. Each of our two lexicons, VERY-NEG and VERY-POS, consists of two lists derived from different values of B, as shown in Tables 2 and 3.

**Table 2:** Negative lexicons: Total number of words (adjectives and adverbs) for each lexicon, and number of words for each class (MN and NMN) in each lexicon.

	Number of words			MN			NMN		
Lexicon	ADJ	ADV	Total	ADJ	ADV	Total	ADJ	ADV	Total
VERY-NEG B = 1	11670	2790	14460	4178	1092	5270	7492	1698	9190
VERY-NEG B = 2	11557	2771	14328	4966	1266	6232	6591	1505	8096
SO-CAL NP1	2826	876	3702	189	62	251	2637	814	3451
SO-CAL NP2	2826	876	3702	536	135	671	2290	741	3031
SO-CAL NP3	2826	876	3702	1080	289	1369	1746	587	2333
SO-CAL NP4	2826	876	3702	1576	429	2005	1250	447	1697
SentiWords NP1	13425	2811	16236	156	4	160	13269	2807	16076
SentiWords NP2	13425	2811	16236	1132	24	1156	12293	2787	15080
SentiWords NP3	13425	2811	16236	4016	189	4205	9409	2622	12031
SentiWords NP4	13425	2811	16236	7612	540	8152	5813	2271	8084

**Table 3:** Positive lexicons: Total number of words (adjectives and adverbs) for each lexicon, and number of words for each class (MP and NMP) in each lexicon.

	Number of words			MP			NMP		
Lexicon	ADJ	ADV	total	ADJ	ADV	Total	ADJ	ADV	Total
VERY-POS B = 1	11402	2769	14171	4721	1163	5884	6681	1606	8287
VERY-POS B = 2	11472	2772	14244	5753	1339	7092	5719	1433	7152
SO-CAL PP1	2826	876	3702	239	75	314	2587	801	3388
SO-CAL PP2	2826	876	3702	512	167	679	2314	709	3023
SO-CAL PP3	2826	876	3702	835	292	1127	2155	628	2783
SO-CAL PP4	2826	876	3702	1250	447	1697	1576	429	2005
SentiWords NP1	13425	2811	16236	130	13	143	13295	2798	16093
SentiWords NP2	13425	2811	16236	581	34	615	12844	2777	15621
SentiWords NP3	13425	2811	16236	2418	250	2668	11007	2561	13568
SentiWords NP4	13425	2811	16236	5813	2271	8084	7612	540	8152

As our main objective is to compare VERY-NEG and VERY-POS with other popular handcrafted lexical resources, we describe two existing lexicons in the next subsections.

#### SO-CAL lexicon

SO-CAL was described in [11]. The authors created their dictionary manually since they believe that the overall accuracy of lexicon-based sentiment analysis mainly relies on the quality of those resources. The lexicon was built with content words, namely adjectives, adverbs, nouns and verbs, adding sentiment scores between -5 and +5. The Negative sign (-) refers to negative polarity while the positive sign (+) indicates positive polarity, and any semantically neutral word has zero score. This dictionary is used for sentiment analysis by means of a lexicon-based classification algorithm, similar to that defined above in Figs 2 and 3.

#### SentiWords lexicon

Sentiwords is a sentiment lexicon derived from SentiWordNet using the method described in [43]. It contains more than 16,000 words provided with a sentiment score between -1 (very negative) and +1 (very positive). The words in this lexicon are arranged with WordNet synsets, that include adjectives, nouns, verbs and adverbs.

### The evaluated lexicons

In order to compare the lexicons, SO-CAL and SentiWords were prepared in the same way as VERY-NEG and VERY-POS.

As far as SentiWords was concerned, we modified the range of values in order to make it similar to that of SO-CAL, make the two lexicons comparable. For this purpose, we multiplied polarity scores by 5 to provide polarity values within the -5 to 5 range, instead of -1 to 1,exactly in the same way as has been done in [11].

To make sure that the comparison of the performance of the lexicons will be fair, SO-CAL and SentiWords were divided into several lexicons. More precisely, they were split into two scales, Negative Polarity (NP) and Positive Polarity (PP), with four partitions on each scale, according to the polarity scores. The different lexicons derived from the original SO-CAL and SentiWords are defined as follows:

- **NP1**: The MN class consists of the words that are ranked as -4 and -5. The other class (NMN) contains the rest of the words.
- **NP2**: MN consists of the words that are rated as -3, -4 and -5. NMN contains the rest of the words.
- **NP3**: MN consists of the words that carry all negative ranks except -1, while the rest were considered as belonging to the class NMN.
- **NP4**: MN class consists of words with all negative ranks from -5 to -1, while NMN class contains all the words from positive ranks: from +1 to +5.
- **PP1**: The MP class consists of the words that are ranked as -4 and +5. The second class (NMP) contains the rest of the words.
- **PP2**: MP consists of the words that are rated as +3, +4 and +5. NMP contains the rest of the words.
- **PP3**: MP consists of the words that carry all positive ranks except +1, while the rest were considered as belonging to the NMP class.
- **PP4**: MP class consists of words with all positive ranks (from +5 to +1), while NMP class contains all the words with negative ranks: from -1 to -5.

Tables 2 and 3 show the total number of words of all the evaluated partitions of lexicons. The tables also include the number of words of each lexicon partition for each class (MN, NMN, MP, NMP).

### Test datasets

Table 4 describes the five datasets that were used to evaluate the performance of the lexicons in the sentiment classification task.

**Table 4:** Size of the five test datasets and the total number of reviews in each class (MN *vs.* NMN) and (MP *vs.* NMP).

Datasets	# of Reviews	MN	NMN	MP	NMP
*Books*	2000	522	1478	731	1269
*DVDs*	2000	530	1470	714	1286
*Electronics*	2000	666	1334	680	1320
*Kitchens*	2000	687	1313	754	1246
*Movies*	50000	14708	35292	14338	35662

#### Multi-domain sentiment dataset

This dataset was used in [44]. It contains product reviews taken from Amazon.com for 4 types of products (domains): Kitchen, Books, DVDs, and Electronics. It is publically available at: https://www.cs.jhu.edu/~mdredze/datasets/sentiment/domain_sentiment_data.tar.gz. The star ratings of the reviews are from 1 to 5 stars. In our experiments, we adopted the scale with five categories. In this case, the borderline separating the MN values from the rest was set to 1, which stands for the MN reviews. The documents in the other four categories were put in the NMN class. According to this borderline value, the MP class was made up of those reviews scored with 5, while the NMP class was built with the rest of reviews.

#### Movie review dataset

This collection of documents, which was reported in [45], consists of 50,000 reviews from IMDB, allowing less than 30 reviews per movie.

The dataset consists of two balanced training and test sets, with 25,000 reviews each http://ai.stanford.edu/~amaas/data/sentiment/.

As we are dealing with an unsupervised method, both the training and test data were integrated in a single corpus. The rating scale is larger than in the previous dataset: it goes from 1 to 10. The borderline variable was set to 2, so MN reviews were assigned values between 1 and 2. The reviews in the other 8 categories were assigned to the class NMN. The same procedure was carried out within the positive scale.

### Evaluation

The lexicons are evaluated on the five collections of scaled reviews by using the classification algorithm explained above in Figs 2 and 3.

Eq 8 defines precision *P*_*neg*_, which is applied to evaluate the classification MN *Vs.* NMN. Similarly, Eq 9 defines precision *P*_*pos*_, which is applied to MP *Vs.* NMP classification.

$$P_{neg}=\frac{trueMN}{trueMN+falseMN}$$ (8)

$$P_{neg}=\frac{trueMP}{trueMP+falseMP}$$ (9)

Eq 10 defines recall *R*_*neg*_, used for MN *Vs.* NMN classification. Eq 11 defines recall *R*_*pos*_, for MP *Vs.* NMP

$$R_{neg}=\frac{trueMN}{trueMN+falseNMN}$$ (10)

$$R_{pos}=\frac{trueMP}{trueMP+falseNMP}$$ (11)

Eqs 12 and 13 are used to compute the f-score, which is the weighted average of the precision and recall.

$$F1_{neg}=2*\frac{P_{neg}*R_{neg}}{P_{neg}+R_{neg}}$$ (12)

$$F1_{pos}=2*\frac{P_{pos}*R_{pos}}{P_{pos}+R_{pos}}$$ (13)

#### Very negative classification (MN vs NMN)

Tables 5, 6 and 7 show the scores (in terms of (P_*neg*_, R_*neg*_, and F1_*neg*_) of the MN and NMN classes for the three lexicons across the four partitions. The experiments were carried out by applying the algorithm described in Fig 2. Tables 5 and 6 summarize the results using the SO-CAL and SentiWords lexicons in all partitions (NP1,NP2, NP3 and NP4). The most interesting finding is that the best F1_*neg*_ has been achieved when using partition NP4 in both lexicons. Table 7 summarizes the results using two versions of our lexicon: the first lexicon was built with borderline value *B* = 1, and the second one with *B* = 2.

**Table 5:** Polarity classification results for all collections with the SO-CAL lexicon, in terms of precision (P_*neg*_), recall (R_*neg*_) and F1_*neg*_ scores for most negative (MN) and other (NMN) class of documents. The best F1_*neg*_ for the *most negative* class in each dataset is highlighted (in bold).

	NP1			NP2			NP3			NP4		
Dataset	P_*neg*_	R_*neg*_	F1_*neg*_	P_*neg*_	R_*neg*_	F1_*neg*_	P_*neg*_	R_*neg*_	F1_*neg*_	P_*neg*_	R_*neg*_	F1_*neg*_
*Books*	0.36	0.06	0.10	0.47	0.13	0.20	0.50	0.26	0.34	0.46	0.50	**0.48**
*DVDs*	0.60	0.10	0.17	0.58	0.18	0.28	0.56	0.31	0.40	0.48	0.51	**0.49**
*Electronics*	0.57	0.13	0.21	0.62	0.20	0.31	0.62	0.29	0.39	0.55	0.49	**0.52**
*Kitchens*	0.59	0.10	0.17	0.64	0.19	0.29	0.66	0.29	0.40	0.57	0.48	**0.52**
*Movies*	0.13	0.03	0.05	0.30	0.14	0.19	0.40	0.30	0.34	0.42	0.55	**0.48**

**Table 6:** Polarity classification results for all collections with the SentiWords lexicon, in terms of precision (P_*neg*_), recall (R_*neg*_) and F1_*neg*_ scores for most negative (MN) and other (NMN) documents. The best F1_*neg*_ for the *most negative* class in each dataset is highlighted (in bold).

	NP1			NP2			NP3			NP4		
Dataset	P_*neg*_	R_*neg*_	F1_*neg*_	P_*neg*_	R_*neg*_	F1_*neg*_	P_*neg*_	R_*neg*_	F1_*neg*_	P_*neg*_	R_*neg*_	F1_*neg*_
*Books*	0.42	0.01	0.02	0.35	0.01	0.03	0.28	0.04	0.07	0.24	0.43	**0.31**
*DVDs*	0.33	0.01	0.01	0.53	0.03	0.06	0.58	0.13	0.22	0.49	0.41	**0.44**
*Electronics*	0.26	0.01	0.01	0.37	0.02	0.03	0.63	0.18	0.28	0.57	0.49	**0.53**
*Kitchens*	0.36	0.01	0.01	0.56	0.01	0.03	0.71	0.17	0.27	0.62	0.45	**0.52**
*Movies*	0.09	0.00	0.00	0.31	0.01	0.01	0.32	0.05	0.08	0.44	0.25	**0.32**

**Table 7:** Polarity classification results for all collections with VERY-NEG lexicon, in terms of precision (P_*neg*_), recall (R_*neg*_) and F1_*neg*_ scores for most negative (MN) and other (NMN) documents. The best F1_*neg*_ for the *most negative* class in each dataset is highlighted (in bold).

	VERY-NEG B = 1			VERY-NEG B = 2		
Dataset	P_*neg*_	R_*neg*_	F1_*neg*_	P_*neg*_	R_*neg*_	F1_*neg*_
*Books*	0.42	0.64	0.51	0.40	0.80	**0.53**
*DVDs*	0.43	0.76	**0.55**	0.88	0.88	0.53
*Electronics*	0.50	0.80	**0.62**	0.45	0.86	0.59
*Kitchen*	0.52	0.70	**0.60**	0.47	0.80	0.59
*Movies*	0.42	0.77	**0.54**	0.39	0.89	**0.54**

**Fig 4:**
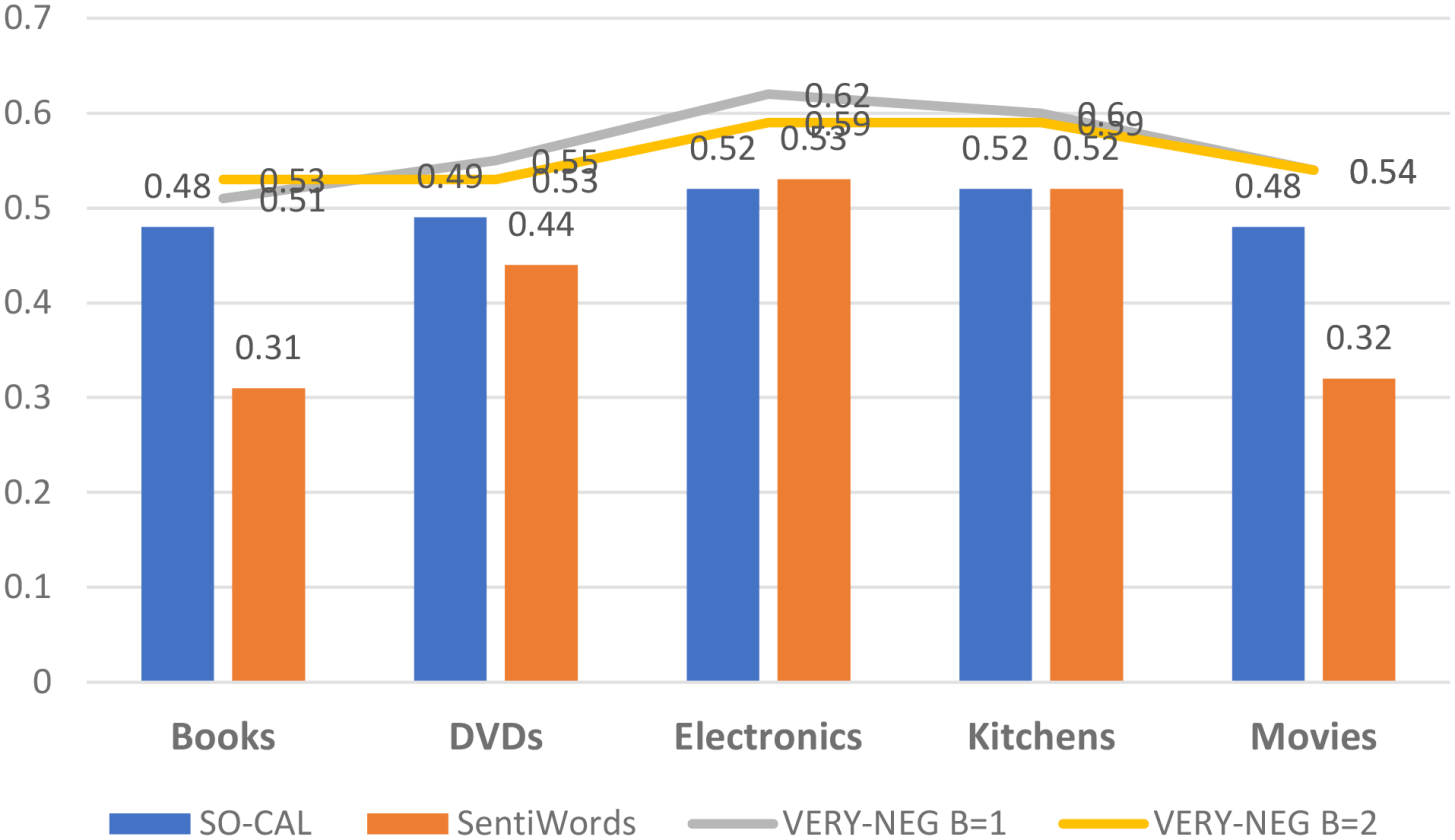
The best performance (F1_*neg*_) obtained by all lexicons on all datasets for identifying most negative documents (MN *vs* NMN).

By comparing the results shown in the three Tables (5, 6 and 7) on the three lexicons, we may make the following observations:

- The best F1_*neg*_ scores in all datasets have been achieved by the two versions of VERY-NEG lexicon. The *B* = 1 version is the best on DVDs, Electronics and Kitchen datasets, while the *B* = 2 version performs better on Books and Movies.
- In all tests, we can observe that the evaluation values for identifying the MN class are low.
- We can also observe in all tests that the best F1_*neg*_ scores were reached using the Electronics and Kitchen datasets, while the worst values were obtained with Movies and Books.
- In general, the behavior of Movies and Books tends to be different from the other datasets.
- The lexicon we proposed, VERY-NEG, consistently outperforms the other lexicons on the five datasets as shown in Fig 4.

#### Very positive classification (MP vs NMP)

Tables 8, 9, and 10 show the scores (in terms of (P_*pos*_, R_*pos*_, and F1_*pos*_) of MP/NMP for the three lexicons across the four partitions. The experiments were carried out by applying the algorithm described above in Fig 3. Tables 8 and 9 show the results obtained using the SO-CAL and SentiWords lexicons. The best F1_*pos*_ scores in both lexicons on all datasets were achieved when partition PP4 was used. Table 10 summarizes the results using two versions of our lexicon again: the one defined with *B* = 1, and the second one with *B* = 2.

**Table 8:** Polarity classification results for all collections with SO-CAL lexicon, in terms of precision (P_*pos*_), recall (R_*pos*_) and F1_*pos*_ scores for most positive (MP) and other (NMP) documents. The best F1_*pos*_ for the *most Positive* class in each dataset is highlighted (in bold).

	PP1			PP2			PP3			PP4		
Dataset	P_*pos*_	R_*pos*_	F1_*pos*_	P_*pos*_	R_*pos*_	F1_*pos*_	P_*pos*_	R_*pos*_	F1_*pos*_	P_*pos*_	R_*pos*_	F1_*pos*_
*Books*	0.61	0.17	0.27	0.54	0.34	0.42	0.52	0.55	0.53	0.41	0.94	**0.57**
*DVDs*	0.66	0.21	0.32	0.58	0.38	0.46	0.54	0.56	0.55	0.41	0.95	**0.58**
*Electronics*	0.54	0.26	0.35	0.51	0.40	0.45	0.49	0.60	0.54	0.38	0.94	**0.54**
*Kitchens*	0.53	0.23	0.32	0.53	0.36	0.43	0.50	0.55	0.52	0.42	0.97	**0.59**
*Movies*	0.75	0.11	0.20	0.60	0.29	0.39	0.52	0.49	0.50	0.35	0.94	**0.51**

**Table 9:** Polarity classification results for all collections with SO-CAL lexicon, in terms of precision (P_*pos*_), recall (R_*pos*_) and F1_*pos*_ scores for most positive (MP) and other (NMP) documents. The best F1_*pos*_ for the *most positive* class in each dataset is highlighted (in bold).

	PP1			PP2			PP3			PP4		
Dataset	P_*pos*_	R_*pos*_	F1_*pos*_	P_*pos*_	R_*pos*_	F1_*pos*_	P_*pos*_	R_*pos*_	F1_*pos*_	P_*pos*_	R_*pos*_	F1_*pos*_
*Books*	0.76	0.06	0.12	0.66	0.13	0.22	0.60	0.38	0.46	0.40	0.93	**0.55**
*DVDs*	0.65	0.07	0.21	0.64	0.13	0.22	0.59	0.38	0.46	0.39	0.92	**0.55**
*Electronics*	0.70	0.11	0.19	0.71	0.19	0.30	0.63	0.41	0.50	0.40	0.93	**0.55**
*Kitchens*	0.61	0.07	0.13	0.63	0.17	0.27	0.65	0.37	0.47	0.43	0.94	**0.59**
*Movies*	0.64	0.01	0.03	0.63	0.05	0.09	0.55	0.27	0.36	0.31	0.95	**0.47**

**Table 10:** Polarity classification results for all collections with VERY-POS lexicon, in terms of precision (P_*pos*_), recall (R_*pos*_) and F1_*pos*_ scores for most positive (MP) and other (NMP) documents. The best F1_*pos*_ for the *most positive* class in each dataset is highlighted (in bold).

	VERY-POS B = 1			VERY-POS B = 2		
Dataset	P_*pos*_	R_*pos*_	F1_*pos*_	P_*pos*_	R_*pos*_	F1_*pos*_
*Books*	0.67	0.55	0.61	0.61	0.67	**0.64**
*DVDs*	0.68	0.49	0.57	0.63	0.61	**0.62**
*Electronics*	0.63	0.42	0.50	0.57	0.52	**0.54**
*Kitchen*	0.63	0.43	0.51	0.60	0.60	**0.60**
*Movies*	0.63	0.41	0.50	0.55	0.58	**0.57**

**Fig 5:**
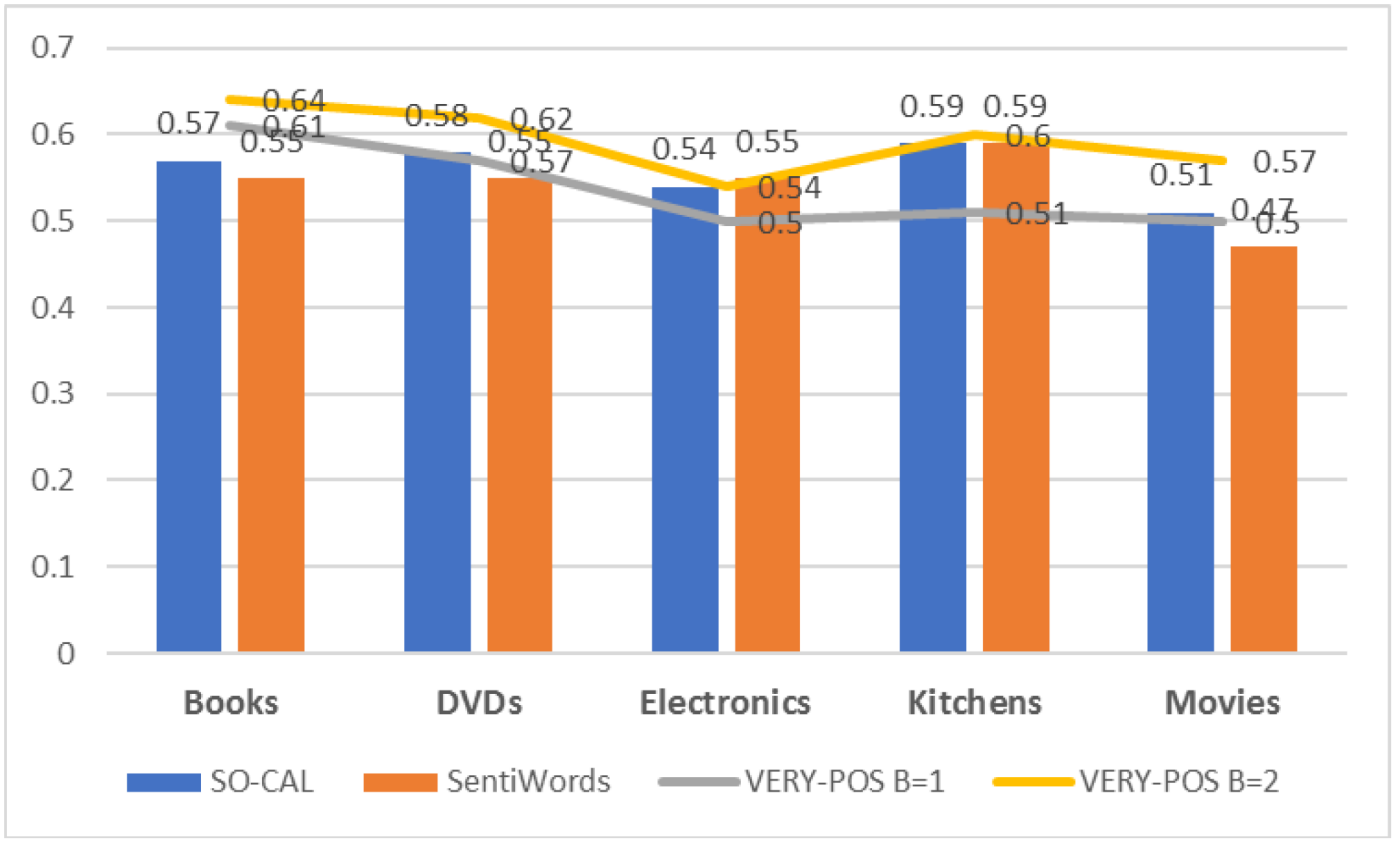
The best performance (F1_*pos*_) obtained by all lexicons on all datasets for identifying the most positive documents.

By comparing the results to differentiate between MP and NMP, we may make the following observations:

- In all datasets, the highest F1_*pos*_ values were reached by the version of VERY-POS lexicon with *B* = 2.
- The evaluation values for identifying the MP class are again low.
- Surprisingly, the highest F1_*pos*_ values were obtained on the Books dataset while the worst scores were on Movies and Electronics. This was not expected because the Electronics dataset was the dataset with the highest scores in identifying the most negative views and the Books was the dataset with the lowest scores.
- The lexicon we proposed, VERY-POS, consistently outperforms the other lexicons on the five datasets as shown in Fig 5.

## Discussion

The low values achieved by the sentiment classification method can be partially explained by the difficulty of the task. The difference between extreme and not extreme is a subjective continuum without clearly defined edges. It is much more difficult to grasp that the difference between negative and positive. Notice that there is a barrier consisting of neutral words between negative and positive values. By contrast, no qualitative borderline can be found between very negative and less negative scores or very positive and less positive scores.

The poor results with the Movies dataset might be due to the fact that films are symbolic objects with an internal plot and, thus, it is natural that a person has a very positive opinion of a plot with many negative elements. The same is true the other way round. This makes sentiment analysis of movies very difficult. As books are also symbolic objects, we are not able to explain why the results of Books do not follow the same tendency as Movies in the MP/MPN task. Finally, a possible explanation for the very poor performance of SO-CAL and SentiWords lexicons in the first three partitions (NP1,NP2,NP3,PP1,PP2 and PP3) might be the unbalanced number of words across the two classes in each case as shown in Tables 2 and 3.

In sum, our automatic strategy for building corpus-based lexicons improves existing manual resources for the task of identifying the extreme opinion.

## Conclusion

The main goal of the current study is to place value on extreme opinions because of their importance in various fields. For this purpose, we have presented a method to automatically build a lexicon of extremely negative and positive words from labeled corpora. Then, we integrated it into a classifier to search for the extreme reviews. Our classifier identifies extreme opinions in two steps. On the one hand, it identifies extremely negative documents from the rest, and on the other, it classifies extremely positive documents from the rest. Our classification algorithm is based on a very basic word-matching scheme to carried out unsupervised sentiment analysis.

Our automatically built lexicons have been compared with handcrafted lexicons, by taking into account some partitions of them. For this purpose, we divide each handcrafted lexicon into partitions depending on the polarity weight of each word. Then, the experiments were carried out on each partition separately.

The results of the experiments show that our lexicons are better suited to identify the extreme opinions than two well-known resources: SO-CALL and SentiWords (a version of SentiWordNet).
